# Synchronous occurrence of small cell lung cancer and primary rectal dedifferentiated liposarcoma with osteosarcomatous differentiation: A rare case report

**DOI:** 10.1097/MD.0000000000035465

**Published:** 2023-09-29

**Authors:** Xiangyu Zheng, Guangfeng Wu, Yongxian Fu, Rui Fan

**Affiliations:** a Department of Pathology, Henan University attached Nanyang first people Hospital, Nanyang, Henan Province, China.

**Keywords:** dedifferentiated liposarcoma, osteosarcoma differentiation, small cell lung cancer, synchronous tumors

## Abstract

**Rationale:**

Rectal dedifferentiated liposarcoma (DDL) and DDL with osteosarcomatous differentiation both are extremely unwonted. In addition, there are no reports of simultaneous DDL with osteosarcoma differentiation with small cell lung cancer (SCLC) to date. Therefore, it is imperative to alert clinicians and pathologists to this extremely rare and instructive synchronous tumor.

**Patient concerns:**

The patient was a 63-year-old male who presented with intermittent hematochezia and a swelling in the anus. Irregular masses were found on computed tomography (CT) examinations of the chest and abdomen respectively.

**Diagnosis:**

The final diagnosis of synchronous occurrence of SCLC and primary rectal DDL with osteosarcomatous differentiation was established by radiological, histological, immunohistochemical and molecular findings.

**Interventions:**

The patient underwent a puncture biopsy of the right lung mass and a complete resection of the rectal mass.

**Outcomes:**

The patient abandoned treatment, and multiple SCLC metastases appeared multiple metastasis 8 months after the operation. In the end, he expired suddenly due to severe cerebral hemorrhage caused by brain SCLC metastasis.

**Lessons:**

DDL with osteosarcoma differentiation is infrequent, and its accurate diagnosis is based on morphology, immunohistochemistry and the necessary molecular tests. In rare cases, DDL occurs concurrently with other malignancies and and will be a challenge for pathologists and clinicians at this time. Accordingly, a comprehensive examination to identify possible synchronous tumors is very important in clinical practice.

## 1. Introduction

Liposarcoma (LPS) is a soft tissue sarcoma consisting of adipose-derived cells of different degrees of differentiation and atypicality.^[1]^ The term “dedifferentiated liposarcoma” (DDL) was initially proposed by Evans in 1979 to describe the morphological progression from well-differentiated liposarcoma (WDL) to nonlipogenic sarcoma.^[2]^ This kind of tumor is most commonly found in the retroperitoneum and accounts for 10% of liposarcomas.^[2]^ Liposarcoma of the gastrointestinal tract is very rare and is mainly found in the stomach, small intestine and colon.^[3]^ LPS of the rectum is even more rare, with merely 2 cases of DDL of the rectum being reported in the English literature.^[4,5]^ DDL can display heterologous differentiation, the most common of which is myogenic differentiation. Approximately 0.6% to 29% of DDL cases are reported to be associated with bone formation, either as metaplastic osteogenesis or osteosarcoma.^[6]^

Small cell lung cancer (SCLC), which accounts for approximately 14% of lung cancers, is a highly aggressive, poorly differentiated, high-grade neuroendocrine malignancy with a poor 5-year survival rate of approximately 6.7%.^[7]^ Synchronous tumors are described as 2 or more distinct primary tumors diagnosed within 6 months of the initial primary tumor being diagnosed.^[8]^ In clinical practice, synchronous malignancy is remarkably unusual, and its diagnosis and treatment are a conundrum for the pathologist and clinician.^[9]^ Herein, we present an extremely unwonted case of synchronous occurrence of SCLC and primary DDL of the rectum with osteosarcomatous differentiation.

## 2. Case presentation

**Figure 1.**
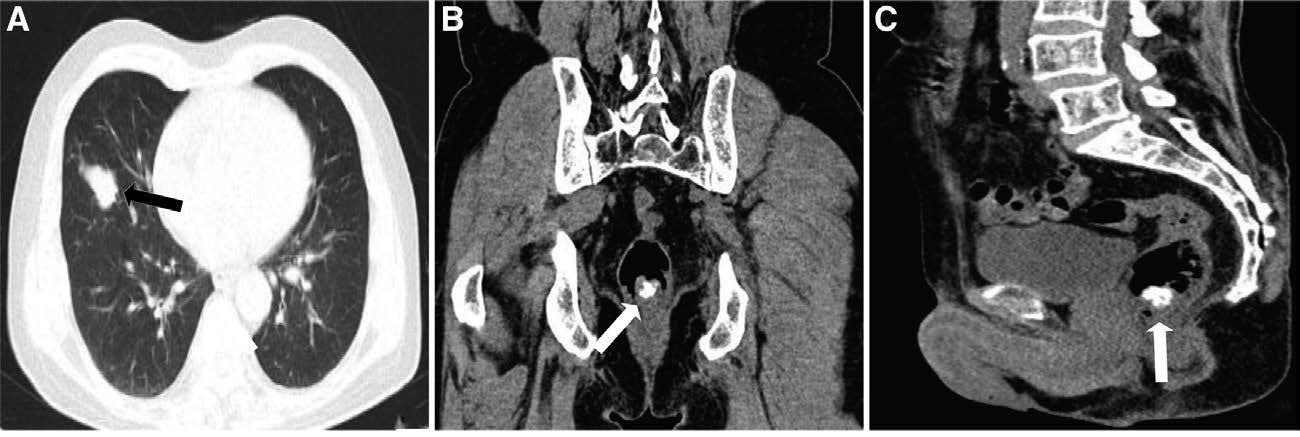
Radiological findings. (A) Thoracic CT indicated irregular nodular soft tissue density shadow in the middle lobe of the right lung (black arrow). (B, C) Axial view and sagittal view of abdominal CT revealed a rectal mass with irregular calcification near the anus (white arrow). CT = computed tomography.

A 63-year-old male patient presented to our hospital because of 6 months of intermittent hematochezia and a gradually increasing swelling in the anus for 3 months. An anal finger examination revealed a hard mass 2 cm above the anus, approximately 30 mm × 20 mm × 20 mm in size, with a tip and good mobility. The patient had no previous medical history of malignant disease and no history of radiation exposure. Thoracic computed tomography (CT) revealed a poorly defined irregular mass in the middle lobe of the right lung (Fig. 1A). Abdominal CT disclosed localized thickened intestinal wall with irregular calcification in the rectum near the anus (Fig. 1B and C). Subsequently, a puncture biopsy of the right lung mass and a complete resection of the rectal mass were performed respectively.

**Figure 2.**
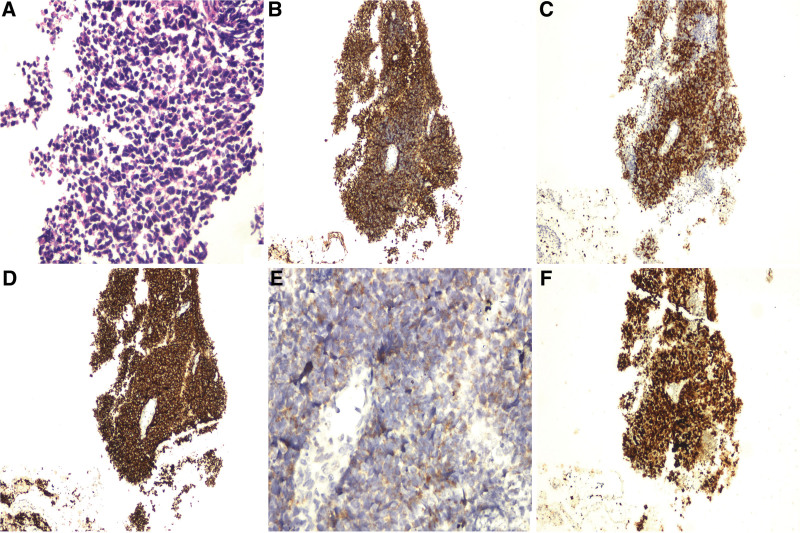
HE and immunohistochemical staining findings of the tumor in the middle lobe of the right lung. (A) High magnification indicated that these small blue roundish or spindle cells were poorly differentiated and had sparse cytoplasm with hyperchromatic stained pretzel-like nuclei (hematoxylin and eosin [H&E], ×400). (B, C) AE1/AE3 and TTF-1 were diffuse positive for these tumor cells (SP × 100). (D, E) Immunohistochemical staining was positive for neuroendocrine markers such as Syn (SP × 100) and CgA (SP × 400). (F) The Ki-67 indexes of these tumor cells were up to 90%. CgA = chromogranin A. Syn = synaptophysin.

Histologically, the tumor of the lung was consisted of infiltrating small blue round or fusiform tumor cells with scant cytoplasm, pepper-salt-like nuclei and high nucleo-cytoplasmic ratio (Fig. 2A). These tumor cells were positive for AE1/AE3, TTF-1 and neuroendocrine markers such as synaptophysin (Syn) and chromogranin A (Fig. 2B–E). The Ki-67 labeling index was up to 90% (Fig. 2F).

**Figure 3.**
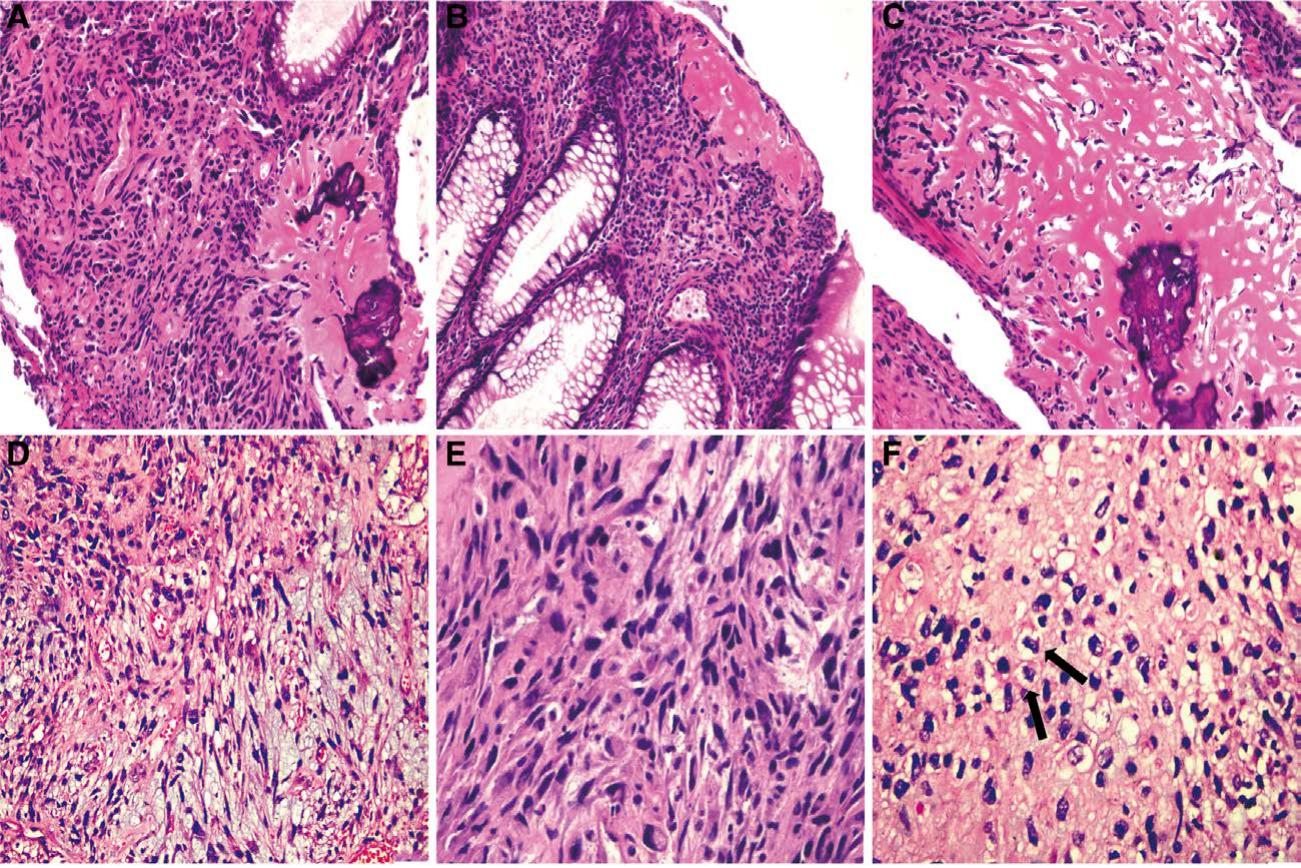
HE staining findings of the rectal tumor. (A) The tumor cells were generally spindle-shaped with marked atypia (left), and areas of osteogenesis were observed (right) (H&E, ×200). (B) These osteogenic components exhibited a minor portion of mature osteoid areas resembling metaplastic osteogenesis (H&E, ×200). (C) Most osteogenic areas displayed osteosarcoma with a high degree of atypia (H&E, ×200). (D) Tumor in focal areas consisted of loosely arranged, slender, spindle-shaped tumor cells in a mucinous background, similar to myxofibrosarcoma-like (H&E, ×200). (E) In a few areas, prominent nuclear pleomorphic tumor cells were observed, resembling undifferentiated sarcoma (H&E, ×400). (F) Scattered lipoblasts with significant pleomorphism can be found (black arrow) (H&E, ×400).

**Figure 4.**
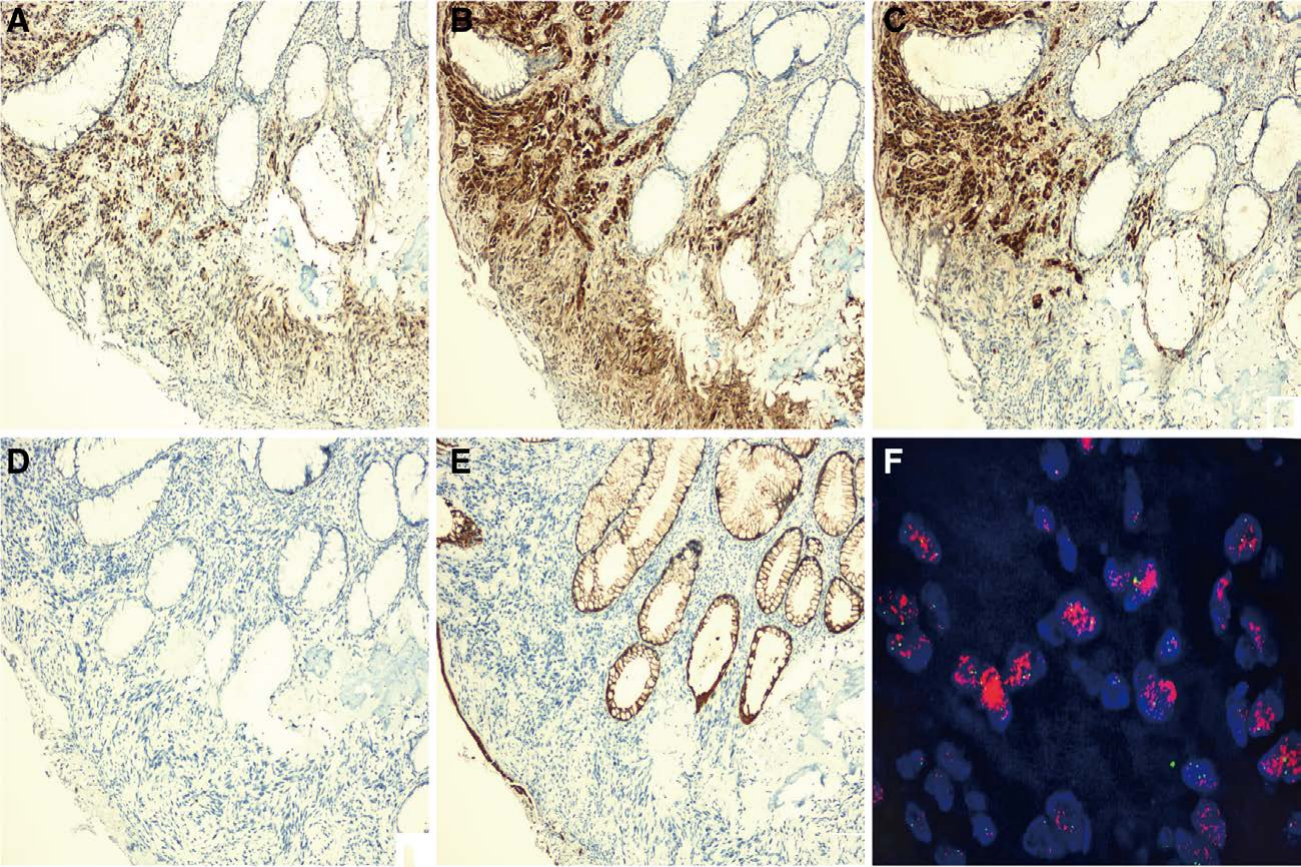
Immunohistochemical and molecular findings of the rectal tumor. (A) MDM2 immunoreactivity in all neoplastic nuclei was observed (SP × 100). (B) Immunohistochemical staining for P16 was diffuse positive in both areas of osteogenesis and high-grade sarcoma without significant bone formation (SP × 100). (C) S100 was diffusely positive in non-osteogenic areas (left), however, it was negatively expressed in osteogenic areas (right) (SP × 100). (D, E) Immunohistochemical staining was negative for HMB45 and AE1/AE3 (SP × 100). (F) FISH revealed high-level amplification of MDM2-gene. FISH = fluorescence in situ hybridization.

Gross examination of the rectal resection mass showed a firm and calcified nodular mass measuring 30 mm × 25 mm × 25 mm. Microscopically, different histological patterns were identified, with the tumor located within the rectal intestinal wall, part of which consisted of osteogenesis with a small group of high-grade mesenchymal-derived non-adipogenic tumors and liposarcomas (Fig. 3A–F). MDM2 and P16 were diffusely positive in areas of osteogenesis (including metaplastic osteogenesis and high-grade osteosarcoma) and in areas of high-grade sarcoma without significant bone formation (Fig. 4A and B). S100 was exclusively and diffusely expressed in non-osteogenic areas (Fig. 4C). HMB45 (Fig. 4D), AE1/AE3 (Fig. 4E), CD117, DOG-1, Desmin, SMA, and H3k27me3 stains were negative. In addition, molecular cytogenetic (fluorescence in situ hybridization) study was performed and amplification of the MDM2 (12q15) loci was detected (Fig. 4F). In light of these radiological, histological, immunohistochemical and molecular findings, a diagnosis of synchronous occurrence of SCLC and primary rectal DDL with osteosarcomatous differentiation was rendered. The patient abandoned treatment and was discharged.

Eight months after the operation, the patient developed multiple SCLC metastases throughout the body. And he finally died suddenly 1 and a half years after surgery due to severe cerebral hemorrhage caused by brain SCLC metastasis.

## 3. Discussion

LPS comprises 20% of malignant mesenchymal tumors and is relatively normal among mesenchymal tumors, with the peak age of onset between 40 and 60 years.^[10]^ DDL represents approximately 10% of all LPS and typically exhibits an atypical lipomatous tumor or WDL conversion to a non-liposarcoma component.^[1]^ DDL commonest histologic presentation is a high-grade sarcoma that is much less aggressive than other types of high-grade pleomorphic sarcomas.^[11]^ Areas of dedifferentiation morphologically resemble undifferentiated sarcoma or myxofibrosarcoma, while heterologous differentiation occurs in approximately 5% to 10% of DDL cases.^[11]^ Heterologous differentiation of DDL can be chondroid, osteoid, or myoid.^[12]^ Based on morphology, most cases of osteoid-like are classified as metaplastic or high-grade osteosarcomatoid ossification, while high-grade osteosarcomatoid ossification cases often have a poor prognosis.^[6]^ The majority of DDL takes place in the retroperitoneum, with other sites including the extremities, parapubic region, and more rarely trunk (including the mediastinum and thorax), the head and neck.^[12]^ DDL of the rectum is extraordinary unwonted, with no more than 2 previously reportorial cases.^[4,5]^ These 3 cases (including the current case) were all male, with the age between 63 and 77 years (average 69 years). Their main symptoms were constipation, hematochezia, abdominal colic and weight loss. The rectal DDL typically ranged from 30 mm to 90 mm in diameter. All of these patients underwent complete surgical removal of the tumor. They had 1 disease-free survival, 1 death from postoperative complications, and 1 death from systemic metastases from other tumors, and no recurrence or metastases from this tumor were reported. Therefore, surgical excision may be sufficient to deal with this anatomical site of DDL.

The main differential diagnosis of this presented case included sarcomatoid carcinoma, synovial sarcoma, malignant melanoma, malignant peripheral nerve sheath tumor, as well as extraskeletal osteosarcoma (ESOS). In the present case, histomorphological, immunohistochemical and molecular findings contributed to the exclusion of the first 4 tumors. However, the identification with ESOS of the rectum can be quite difficult. Although extremely rare, ESOS of the rectum had been reported.^[13]^ Furthermore, MDM2 amplification is not limited to WDL/DDL and had been observed in low grade osteosarcoma, including low grade osteosarcoma indedifferentiated and containing regions resembling pleomorphic sarcoma or high-grade osteosarcoma.^[3]^ In addition, Yamashita et al reported a series of ESOS accompanied by MDM2 amplification.^[3]^ Nevertheless, DDL is much more common than ESOS, and the combination of the histomorphological diversity and the complexity of the immunohistochemical findings of our cases may be more supportive of the diagnosis of DDL.

Radical surgical resection remains the primary treatment strategy for LPS, with radiotherapy (RT) also having some therapeutic value.^[12]^ However, LPS tends to respond poorly to chemotherapeutic agents. Targeted therapy for LPS is a new treatment option. The future of LPS management may be a multimodal approach with a primary focus on radical surgery.^[12]^

SCLC originates in the peribronchial region and accounts for approximately 14% of lung cancer cases. Patients with SCLC frequently have a poor prognosis because these tumors usually have high proliferation rates, early and extensive metastasis, and acquired drug resistance.^[14]^

In our review of the English literatures, DDL had been reported to occur synchronously with renal cell carcinoma, gastrointestinal mesenchymal tumor and gastric cancer, but not with small SCLC.^[15–17]^ This case reminds us that it is essential in clinical practice to perform a comprehensive examination to identify possible synchronous tumors as this could potentially have an impact on the patient treatment and prognosis.

## Acknowledgments

We would like to thank the patient and her family.

## Author contributions

**Conceptualization:** Xiangyu Zheng, Rui Fan.

**Data curation:** Guangfeng Wu.

**Methodology:** Guangfeng Wu, Yongxian Fu.

**Writing – original draft:** Xiangyu Zheng.

**Writing – review & editing:** Xiangyu Zheng, Rui Fan.

## References

[1] Thway K. Well-differentiated liposarcoma and dedifferentiated liposarcoma: an updated review. Semin Diagn Pathol. 2019;36:112–21. 30852045 10.1053/j.semdp.2019.02.006

[2] Gordhandas J Lin G Tipps AMP. Osteosarcomatous divergence in dedifferentiated liposarcoma presenting as a colonic mass. Case Rep Pathol. 2019;2019:8025103. 31380135 10.1155/2019/8025103 PMC6662438

[3] Gajzer DC Fletcher CD Agaimy A. Primary gastrointestinal liposarcoma-a clinicopathological study of 8 cases of a rare entity. Hum Pathol. 2020;97:80–93. 31884085 10.1016/j.humpath.2019.12.004

[4] Tsuruta A Notohara K Park T. Dedifferentiated liposarcoma of the rectum: a case report. World J Gastroenterol. 2012;18:5979–81. 23139616 10.3748/wjg.v18.i41.5979 PMC3491607

[5] Li W Liu Q Mu Y. Dedifferentiated liposarcoma (DDLPS) in the rectum: a case report. J Int Med Res. 2022;50:3000605221102081. 10.1177/03000605221102081 PMC9237925 35751419

[6] Yamashita K Kohashi K Yamada Y. Osteogenic differentiation in dedifferentiated liposarcoma: a study of 36 cases in comparison to the cases without ossification. Histopathology. 2018;72:729–38. 29076540 10.1111/his.13421

[7] Tariq S Kim SY Monteiro de Oliveira Novaes J. Update 2021: management of small cell lung cancer. Lung. 2021;199:579–87. 34757446 10.1007/s00408-021-00486-y

[8] Lim CH Mohamed-Haflah NH Abdullah-Sani MH. Synchronous primary parosteal osteosarcoma and primary mediastinal germ cell tumour with atypical mycobacterial infection - a rare phenomenon: a case report. Malays Orthop J. 2023;17:188–92. 37064628 10.5704/MOJ.2303.023 PMC10103910

[9] Wali L Husain F Shah A. Prostate cancer and sarcoma: challenges of synchronous malignancies. Radiol Case Rep. 2020;15:2303–7. 32983304 10.1016/j.radcr.2020.08.069 PMC7494932

[10] Nakayama S Matsumura K Fukuda A. A case of dedifferentiated liposarcoma of the descending colon. Clin J Gastroenterol. 2023;16:361–5. 36735203 10.1007/s12328-023-01762-5

[11] Sbaraglia M Bellan E Dei Tos AP. The 2020 WHO classification of soft tissue tumours: news and perspectives. Pathologica. 2021;113:70–84. 33179614 10.32074/1591-951X-213 PMC8167394

[12] Thway K Jones RL Noujaim J. Dedifferentiated liposarcoma: updates on morphology, genetics, and therapeutic strategies. Adv Anat Pathol. 2016;23:30–40. 26645460 10.1097/PAP.0000000000000101

[13] Iannaci G Luise R Sapere P. Extraskeletal osteosarcoma: a very rare case report of primary tumor of the colon-rectum and review of the literature. Pathol Res Pract. 2013;209:393–6. 23642451 10.1016/j.prp.2013.03.010

[14] Meijer JJ Leonetti A Airò G. Small cell lung cancer: novel treatments beyond immunotherapy. Semin Cancer Biol. 2022;86(Pt 2):376–85. 35568295 10.1016/j.semcancer.2022.05.004

[15] Beltran E Garcia-Robledo JE Rodríguez-Rojas LX. Clear cell renal carcinoma synchronous with dedifferentiated liposarcoma: a case report and review of the literature. J Med Case Rep. 2020;14:4. 31915049 10.1186/s13256-019-2320-4 PMC6950918

[16] Diamantis A Samara AA Baloyiannis I. Gastrointestinal Stromal Tumor (GIST) and synchronous intra-abdominal liposarcoma: a report of two rare cases and literature review. Int J Surg Oncol. 2021;2021:2626635. 34518784 10.1155/2021/2626635 PMC8434899

[17] Kobayashi H Ohashi R Ujita M. Synchronous occurrence of advanced gastric carcinoma with retroperitoneal liposarcoma: A Case Report. Am J Case Rep. 2022;23:e934586. 34996885 10.12659/AJCR.934586 PMC8754007

